# Cladieunicellins K and L, New Eunicellin-Based Diterpenoids from an Octocoral *Cladiella* sp

**DOI:** 10.3390/ijms141121781

**Published:** 2013-11-04

**Authors:** Fu-Yuan Shih, Tsung-Hung Chen, Mei-Chin Lu, Wu-Fu Chen, Zhi-Hong Wen, Yueh-Hsiung Kuo, Ping-Jyun Sung

**Affiliations:** 1 Department of Neurosurgery, Kaohsiung Chang Gung Memorial Hospital and Chang Gung University College of Medicine, Kaohsiung 833, Taiwan; E-Mails: 8902055@cgmh.org.tw (F.-Y.S.); ma4949@adm.cgmh.org.tw (W.-F.C.); 2 Graduate Institute of Marine Biotechnology, National Dong Hwa University, Pingtung 944, Taiwan; E-Mails: a610162002@gmail.com (T.-H.C.); jinx6609@nmmba.gov.tw (M.-C.L.); 3 National Museum of Marine Biology and Aquarium, Pingtung 944, Taiwan; 4 Department of Marine Biotechnology and Resources and Asia-Pacific Ocean Research Center, National Sun Yat-sen University, Kaohsiung 804, Taiwan; E-Mail: wzh@mail.nsysu.edu.tw; 5 Department of Chinese Pharmaceutical Sciences and Chinese Medicine Resources, China Medical University, Taichung 404, Taiwan; 6 Department of Biotechnology, Asia University, Taichung 413, Taiwan; 7 Graduate Institute of Natural Products, Kaohsiung Medical University, Kaohsiung 807, Taiwan; 8 Chinese Medicine Research and Development Center, China Medical University Hospital, Taichung 404, Taiwan

**Keywords:** eunicellin, *Cladiella*, cladieunicellin, cytotoxicity

## Abstract

Two new eunicellin-based diterpenoids, cladieunicellins K (**1**) and L (**2**), were isolated from an octocoral *Cladiella* sp. The structures of **1** and **2** were elucidated by spectroscopic methods and **2** exhibited moderate cytotoxicity towards the MOLT-4 human leukemia.

## 1. Introduction

**Scheme 1:**
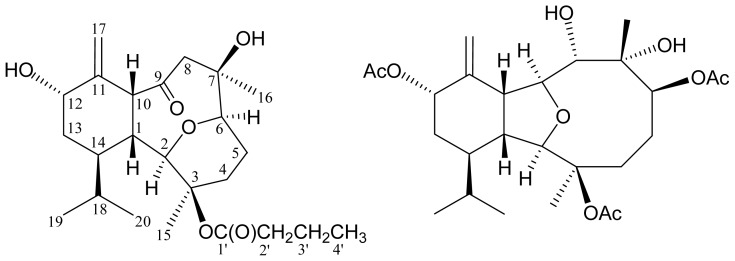
The structures of cladieunicellins K (**1**) and L (**2**).

Eunicellin-based diterpenoids, such as eleutherobin, have been proven to be potential anticancer agents [1]. In our continuing research on the chemical constituents of octocorals belonging to the genus *Cladiella* collected off the waters of Taiwan and Indonesia, various eunicellin-related analogues have been isolated [2–7]. Recently, two new eunicellin-type diterpenoids, cladieunicellins K (**1**) and L (**2**) were isolated from an octocoral identified as *Cladiella* sp. (Scheme 1). In this paper, we describe the isolation, structure determination and cytotoxicity of eunicellins **1** and **2**.

## 2. Results and Discussion

Cladieunicellin K (**1**) was isolated as colorless oil and its molecular formula was established as C_24_H_38_O_6_ (6° of unsaturation) by the HRESIMS at *m*/*z* 445.2564 (calcd for C_24_H_38_O_6_Na, 445.2566). The IR absorptions at υ_max_ 3388 (broad) and 1736 cm^−1^ revealed the presence of hydroxy and carbonyl functionalities. The ^13^C spectrum of **1** showed 24 carbon signals (Table 1), which were assigned with the assistance of the DEPT spectrum to five methyls, six sp^3^ methylenes, an sp^2^ methylene, seven sp^3^ methines (including three oxymethines), two sp^3^ oxygenated quaternary carbons and three sp^2^ quaternary carbons (including two carbonyls). The ^13^C resonances at δ_C_ 213.8 and 172.1 demonstrated the presence of a ketonic carbonyl and an ester carbonyl, respectively. The ester carbonyl was identified as an *n*-butyrate carbonyl by the presence of seven contiguous protons at δ_H_ 1.01 (3H, t, *J* = 7.6 Hz), 1.72 (2H, sext, *J* = 7.6 Hz) and 2.36 (2H, t, *J* = 7.6 Hz). From the ^13^C NMR data, an exocyclic carbon-carbon double bond was deduced from the signals at δ_C_ 116.6 (CH_2_) and 144.4 (C), and confirmed by two olefin proton signals at δ_H_ 4.86 (1H, d, *J* = 1.6 Hz) and 5.12 (1H, d, *J* = 1.6 Hz) in the ^1^H NMR spectrum. Comparison of the ^13^C NMR and DEPT spectra with the molecular formula indicated that there must be two exchangeable protons, requiring the presence of two hydroxy groups. From the above data, **1** had to be tricyclic to account for the three degrees of unsaturation.

From the ^1^H–^1^H COSY spectrum of **1** (Table 1), the spin systems of H_2_-4/H_2_-5/H-6 and H-10/H-1 were differentiated. These data, together with the HMBC correlations among H-1/C-3, -9, -10; H-2/C-1, -3, -10; H_2_-4/C-2, -3, -5, -6; H_2_-5/C-3, -4, -6, -7; H-6/C-7, -8; H-8/C-6, -7, -9; and H-10/C-1, -9, established the connectivity from C-1 to C-10 in the 10-membered ring (Table 1). The 1-isopropyl-4-methylenecyclohexane ring, which is fused to the 10-membered ring at C-1 and C-10, was elucidated by the ^1^H–^1^H COSY correlations between H-1/H-14/H_2_-13/H-12 and H-14/H-18/H_3_-19 (H_3_-20) and by the HMBC correlations between H-1/C-14; H-2/C-14; H-10/C-11, -12, -14, -17; H_2_-13/C-1; and H_2_-17/C-10. The isopropyl group was positioned at C-14 from the HMBC correlations between H-1/C-14; H-18/C-13; and by the mutual and common HMBC correlations: H_3_-19/C-14, -18, -20; and H_3_-20/C-14, -18, -19. An exocyclic carbon-carbon double bond at C-11 was confirmed by the HMBC correlations between H_2_-17/C-10, -11, -12. The ether bridge between C-2 and C-6 was supported by the HMBC correlations between H-2/C-6 and H-6/C-2. The hydroxy proton signal at δ_H_ 6.02 was revealed by its ^1^H–^1^H COSY and HMBC correlations to δ_H_ 4.25 (H-12) and δ_C_ 70.3 (CH-12), respectively, indicating its attachment to C-12. The location of a hydroxy group at C-7, an oxygenated quaternary carbon, was confirmed by the HMBC correlations between a hydroxy proton at δ_H_ 4.59 and C-7, -8 and C-16. Thus, the remaining *n*-butyrate group was at C-3, an oxygenated quaternary carbon which bonded to the C-15 tertiary methyl and was confirmed by the HMBC correlations between H_3_-15/C-2, -3, -4.

**Scheme 2:**
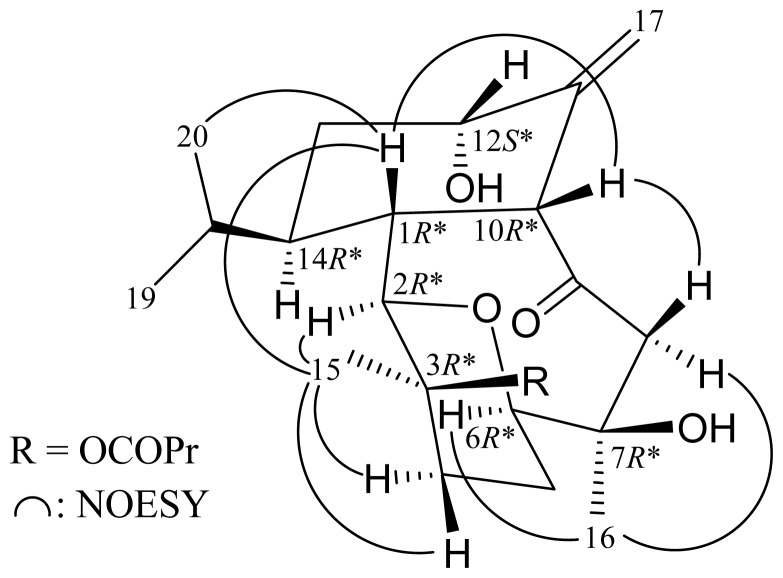
Key NOESY correlations of **1**.

Most naturally occurring eunicellin analogues from soft corals belonging to the genus *Cladiella* have H-1 and H-10 in the β-orientation [8]. The relative configuration of **1** was elucidated mainly from a NOESY spectrum (Scheme 2) and that obtained from vicinal proton coupling constant analysis. In the NOESY experiment, H-1 correlated with H-10 and H_3_-20, but no correlation was found between H-10 and H_3_-20, indicating that H-1 was β-oriented and positioned on the axial direction; and H-10 and the isopropyl group were β-oriented and positioned on the equatorial directions in the cyclohexane ring of **1**. The coupling constants between H-12 and C-13 methylene protons (*J* = 2.8, 2.8 Hz) indicated that H-12 was positioned on equatorial direction and possessed a β-orientation in the cyclohexane ring of **1**. No coupling constant was detected between H-1 and H-2, indicating the dihedral angle between H-1 and H-2 is 90° and H-2 should be α-oriented. The C-15 methyl showed correlations with H-1, H-2 and H-4α/β, but not with H-10, demonstrating the *n*-butyrate group at C-3 was β-oriented. It was found that one of the methylene protons at C-8 (δ_H_ 2.79) exhibited a correlation with H-10, and, therefore, it was assigned as H-8 β, and the other C-8 proton (δ_H_ 2.08) as H-8α. The correlations between H_3_-16/H-8α and H-6/H_3_-16, suggested the α-orientation of Me-16 and H-6. Based on the above findings, the structure of **1** was elucidated and the chiral carbons for **1** were assigned as 1R*, 2R*, 3R*, 6R*, 7R*, 10R*, 12S* and 14R*.

Cladieunicellin L (**2**) was isolated as colorless oil that gave a molecular ion [M + Na]^+^ at *m*/*z* 519.2567 in the HRESIMS, indicating the molecular formula C_26_H_40_O_9_ (calcd for C_26_H_40_O_9_Na, 519.2570) and implying seven degrees of unsaturation. The IR spectrum of **2** showed bands at υ_max_ 3458 (broad) and 1731 cm^−1^, consistent with the presence of hydroxy and ester groups. Comparison of the ^13^C and DEPT spectral data with the molecular formula indicated that there must be two exchangeable protons, which required the presence of two hydroxy groups. Based on the ^1^H and ^13^C NMR spectra (Table 2), compound **2** was found to possess an exocyclic carbon-carbon double bond (δ_H_ 5.20, 2H, br s; δ_C_ 117.2, CH_2_; 143.1, C) and three acetoxy (δ_H_ 2.07, 2.09, 2.10, each 3H × s, methyls; δ_C_ 21.4, 21.6, 22.4, 3 × methyls; δ_C_ 169.5, 170.4, 171.9, 3 × carbonyls) groups. From the above findings, metabolite **2** was established to be a tricyclic diterpenoid. In addition, a suite of resonances of proton signals at δ_H_ 2.30 (1H, ddd, *J* = 10.4, 7.2, 1.2 Hz), 3.71 (1H, d, *J* = 1.2 Hz), 4.03 (1H, dd, *J* = 9.2, 6.4 Hz) and 3.35 (1H, dd, *J* = 7.2, 6.4 Hz), and carbon signals at δ_C_ 44.3 (CH), 91.3 (CH), 82.5 (CH) and 51.0 (CH), indicated the presence of a tetrahydrofuran moiety.

From the ^1^H–^1^H COSY spectrum of **2** (Table 2), it was possible to identify the spin systems among H-1/H-2, H_2_-4/H_2_-5/H-6, H-8/H-9/H-10/H-1/H-14/H_2_-13/H-12 and H-14/H-18/H_3_-19 (H_3_-20). These data, together with the key HMBC correlations between protons and quaternary carbons of **2** (Table 2), such as H-2, H_2_-4, H_2_-5, H_3_-15/C-3; H_2_-5, H-6, H_3_-16/C-7; and H-9, H-10, H_2_-17/C-11, permitted elucidation of the carbon skeleton. The ether bridge between C-2 and C-9 was supported by the HMBC correlations between H-2/C-9 and H-9/C-2. The locations of acetoxy groups in **2** were confirmed by the HMBC correlations between H-6 (δ_H_ 5.73) and H-12 (δ_H_ 5.46) and the acetate carbonyls at δ_C_ 171.9 and 170.4, respectively. The hydroxy group at C-7, an oxygenated quaternary carbon, was elucidated by the HMBC correlations between a hydroxy proton at δ_H_ 2.36 and C-7, -8 and C-16. The oxymethine proton signal at δ_H_ 3.49 was revealed by its ^1^H–^1^H COSY correlation to δ_H_ 4.03 (H-9) and 2.09 (OH-8), indicating a hydroxy group at C-8. Thus, the remaining acetate group in **2** should be positioned at C-3.

**Scheme 3:**
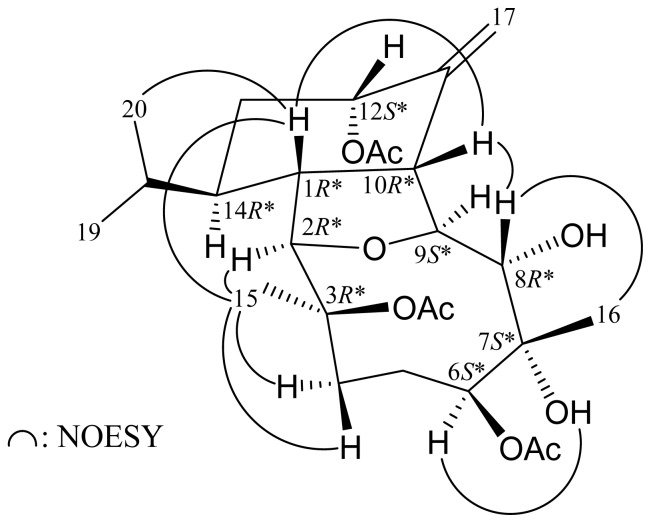
Key NOESY correlations of **2**.

The chiral carbons in the cyclohexane ring of **2** were found to possess the same relative configurations (1R*, 10R*, 12S* and 14R*) as those of **1** by its NOESY correlations (Scheme 3) and vicinal proton constants analysis. The dihedral angle between H-1 and H-2 was inferred to be approximately 90° by a small coupling constant (*J* = 1.2 Hz) between these two protons. Moreover, H-2 should be α-oriented. H-8 exhibited correlations with H-10 and H_3_-16, but not with H-9, suggesting that H-9 and the hydroxy groups at C-7 and C-8 were α-oriented. H-6 correlated with 7-OH, indicating the 6-acetoxy group was β-oriented. H_3_-15 showed correlations with H-1, H-2 and H-4α/β, but not with H-10, demonstrating that the 3-acetoxy group was β-oriented. Based on the above findings, the structure of **2** was elucidated and the chiral carbons for **2** were assigned as 1R*, 2R*, 3R*, 6S*, 7S*, 8R*, 9S*, 10R*, 12S* and 14R*.

Cytotoxicity of compounds **1** and **2** toward HL-60 (human promyelocytic leukemia) and MOLT-4 (human acute T lymphoblastic leukemia) cells was studied, and the results are shown in Table 3. These data showed that cladieunicellin L (**2**) exhibited moderate cytotoxicity towards the MOLT-4 cells.

## 3. Experimental Section

### 3.1. General Experimental Procedures

Optical rotations were measured at a Jasco P-1010 digital polarimeter (Japan Spectroscopic Corporation, Tokyo, Japan). Infrared spectra were recorded on a Varian Diglab FTS 1000 FT-IR spectrometer (Varian Inc., Palo Alto, CA, USA); peaks are reported in cm^−1^. NMR spectra were recorded on a Varian Mercury Plus 400 NMR spectrometer (Varian Inc., Palo Alto, CA, USA) using the residual CHCl_3_ signal (δ_H_ 7.26 ppm) as the internal standard for ^1^H NMR and CDCl_3_ (δ_C_ 77.1 ppm) for ^13^C NMR. Coupling constants (*J*) are given in Hz. ESIMS and HRESIMS were recorded using a Bruker APEX II mass spectrometer (Bruker, Bremen, Germany). Column chromatography was performed on silica gel (230–400 mesh, Merck, Darmstadt, Germany). TLC was carried out on precoated Kieselgel 60 F_254_ (0.25 mm, Merck, Darmstadt, Germany); spots were visualized by spraying with 10% H_2_SO_4_ solution followed by heating. The normal phase HPLC (NP-HPLC) was performed using a system comprised of a Hitachi L-7110 pump (Hitachi Ltd., Tokyo, Japan) and a Rheodyne 7725 injection port (Rheodyne LLC, Rohnert Park, CA, USA). Two normal phase columns (Supelco Ascentis^®^ Si Cat #:581515-U, 25 cm × 21.2 mm, 5 μm; 581514-U, 25 cm × 10 mm, 5 μm, Sigma-Aldrich. Com., St. Louis, MO, USA) were used for NP-HPLC. The reverse phase HPLC (RP-HPLC) was performed using a system comprised of a Hitachi L-7100 pump (Hitachi Ltd., Tokyo, Japan), a Hitachi L-2455 photodiode array detector (Hitachi Ltd., Tokyo, Japan), a Rheodyne 7725 injection port (Rheodyne LLC, Rohnert Park, CA, USA) and a reverse column (Varian Polaris C18-A, 250 mm × 10 mm, 5 μm; Varian Inc., Palo Alto, CA, USA) were used for RP-HPLC.

### 3.2. Animal Material

Specimens of the octocoral *Cladiella* sp. [9] were collected by hand using scuba equipment off the coast of Penghu Archipelago, Taiwan in September 2011, and stored in a freezer (−20 °C) until extraction. A voucher specimen (NMMBA-TWSC-11011) was deposited in the National Museum of Marine Biology and Aquarium, Taiwan.

### 3.3. Extraction and Isolation

Specimens of the soft coral *Cladiella* sp. (wet weight 1.25 kg, dry weight 457 g) were minced and extracted with ethyl acetate (EtOAc). The EtOAc extract left after removal of the solvent (12.4 g) was separated by silica gel and eluted using *n*-hexane/EtOAc in a stepwise fashion from 100:1–pure EtOAc to yield 16 fractions A–P. Fraction N was chromatographed on silica gel, using a mixture of *n*-hexane and acetone in a stepwise fashion from 6:1–pure acetone to obtain 15 subfractions N1–N15. Fraction N4 was repurified by NP-HPLC, using a mixture of *n*-hexane and acetone (10:1, flow rate: 2.0 mL/min) to yield seven subfractions N4A–N4G. Fraction N4E was further separated by RP-NPLC, using a mixture of methanol and water (8:2, flow rare: 1.0 mL/min) to yield cladieunicellin K (**1**) (1.1 mg, *t*_R_ = 24 min). The residue of fraction N11 was separated by NP-HPLC, using a mixture of *n*-hexane and acetone (3:1, flow rate: 2.0 mL/min) to obtain cladieunicellin L (**2**) (2.8 mg, *t*_R_ = 110 min).

Cladieunicellin K (**1**): colorless oil; ${\left[\alpha \right]}_{\text{D}}^{22}-14\;(c\;0.06,\;{\text{CHCl}}_{3})$; IR (neat) ν_max_ 3388, 1736 cm^−1^; ^1^H (400 MHz, CDCl_3_) and ^13^C (100 MHz, CDCl_3_) NMR data, see Table 1; ESIMS: *m*/*z* 445 (M + Na)^+^; HRESIMS: *m*/*z* 445.2564 (calcd for C_24_H_38_O_6_Na, 445.2566).

Cladieunicellin L (**2**): colorless oil; ${\left[\alpha \right]}_{\text{D}}^{22}-10\;(c\;0.17,\;{\text{CHCl}}_{3})$; IR (neat) ν_max_ 3458, 1731 cm^−1^; ^1^H (400 MHz, CDCl_3_) and ^13^C (100 MHz, CDCl_3_) NMR data, see Table 2; ESIMS: *m*/*z* 519 (M + Na)^+^; HRESIMS: *m*/*z* 519.2567 (calcd for C_26_H_40_O_9_Na, 519.2570).

### 3.4. Cytotoxicity Testing

Cytotoxicity of compounds **1** and **2** was assayed with a modification of the MTT [3-(4,5-dimethylthiazol-2-yl)-2,5-diphenyltetrazolium bromide] colorimetric method according to previously described procedures [10,11].

## 4. Conclusions

Two new eunicellin-based diterpenoids, cladieunicellins K (**1**) and L (**2**), were isolated from the soft coral *Cladiella* sp. Compound **2** showed moderate cytotoxicity toward the MOLT-4 leukemia. Thus, compound **2** could be promising a bioactive agent and may warrant further biomedical investigation.

## Figures and Tables

**Table 1:** ^1^H (400 MHz, CDCl_3_) and ^13^C (100 MHz, CDCl_3_) NMR data, ^1^H–^1^H COSY and HMBC correlations for cladieunicellin K (**1**).

Position	δ_H_ (*J* in Hz)	δ_C_, Multiple	^1^H–^1^H COSY	HMBC
1	2.30 dd (12.0, 4.8)	55.5, CH	H-10, H-14	C-3, -9, -10, -14
2	3.96 s	77.5, CH	n.o. a	C-1, -3, -6, -10, -14, -15
3		81.2, C		
4	2.98 ddd (13.6, 4.0, 2.0) 1.42 m	27.5, CH_2_	H_2_-5	C-2, -3, -5, -6
5	1.68 m; 1.34 m	20.5, CH_2_	H_2_-4, H-6	C-3, -4, -6, -7
6	3.86 dd (12.0, 6.4)	80.2, CH	H_2_-5	C-2, -7, -8
7		85.8, C		
8	2.79 d (12.0) 2.08 d (12.0)	47.7, CH_2_		C-6, -7, -9, -16
9		213.8, C		
10	4.41 d (4.8)	57.5, CH	H-1	C-1, -9, -11, -12, -14, -17
11		144.4, C		
12	4.25 ddd (10.4, 2.8, 2.8)	70.3, CH	H_2_-13, OH-12	n.o.
13	2.02 m; 1.26 m	34.4, CH_2_	H-12, H-14	C-1, -11, -12
14	2.23 dddd (12.0, 12.0, 2.4, 2.4)	32.0, CH	H-1, H_2_-13, H-18	n.o.
15	1.55 s	23.4, CH_3_		C-2, -3, -4
16	1.16 s	22.9, CH_3_		C-6, -7, -8
17	5.12 d (1.6); 4.86 d (1.6)	116.6, CH_2_		C-10, -11, -12
18	1.98 m	27.0, CH	H-14, H_3_-19, H_3_-20	C-13, -19
19	1.02 d (6.8)	21.6, CH_3_	H-18	C-14, -18, -20
20	0.70 d (6.8)	14.6, CH_3_	H-18	C-14, -18, -19
3-OCOCH_2_CH_2_CH_3_		172.1, C		
1′ 2′ 3′ 4′	2.36 t (7.6)	37.7, CH_2_	H_2_-3′	C-1′, -3′, -4′
	1.72 sext (7.6)	18.6, CH_2_	H_2_-2′, H_3_-4′	C-1′, -2′, -4′
	1.01 t (7.6)	13.7, CH_3_	H_2_-3′	C-2′, -3′
7-OH	4.59 s			C-7, -8, -16
12-OH	6.02 d (10.4)		H-12	C-12

a n.o. = not observed.

**Table 2:** ^1^H (400 MHz, CDCl_3_) and ^13^C (100 MHz, CDCl_3_) NMR data, ^1^H–^1^H COSY and HMBC correlations for cladieunicellin L (**2**).

Position	δ_H_ (*J* in Hz)	δ_C_, Multiple	^1^H–^1^H COSY	HMBC
1	2.30 ddd (10.4, 7.2, 1.2)	44.3, CH	H-2, H-10, H-14	C-9, -10, -13, -14
2	3.71 d (1.2)	91.3, CH	H-1	C-1, -3, -9, -10, -14, -15
3		86.3, C		
4	2.51 dd (14.8, 8.4); 1.99 m	34.6, CH_2_	H_2_-5	C-2, -3, -5, -6, -15
5	1.63 m; 1.49 m	28.5, CH_2_	H_2_-4, H-6	C-3, -4, -6, -7, -16
6	5.73 dd (6.8, 1.6)	81.7, CH	H_2_-5	C-4, -5, -7, -16, C=O
7		78.1, C		
8	3.49 dd (10.8, 9.2)	79.7, CH	H-9, OH-8	C-9, -10
9	4.03 dd (9.2, 6.4)	82.5, CH	H-8, H-10	C-2, -8, -11
10	3.35 dd (7.2, 6.4)	51.0, CH	H-1, H-9	C-1, -8, -9, -11, -12, -14, -17
11		143.1, C		
12	5.46 dd (4.8, 2.8)	73.4, CH	H_2_-13	C-10, -14, -17, C=O
13	1.95 m; 1.36 m	28.8, CH_2_	H-12, H-14	C-12, -14
14	1.67 m	37.2, CH	H-1, H_2_-13, H-18	
15	1.43 s	22.9, CH_3_		C-2, -3, -4
16	1.30 s	18.5, CH_3_		C-6, -7, -8
17	5.20 br s	117.2, CH_2_		C-10, -11, -12
18	1.81 m	28.7, CH	H-14, H_3_-19, H_3_-20	n.o. a
19	0.96 d (6.8)	21.8, CH_3_	H-18	C-14, -18, -20
20	0.82 d (6.8)	15.7, CH_3_	H-18	C-14, -18, -19
3-OAc	2.10 s	169.5, C 22.4, CH_3_		C=O
6-OAc	2.09 s	171.9, C 21.6, CH_3_		C=O
12-OAc	2.07 s	170.4, C 21.4, CH_3_		C=O
7-OH	2.36 s			C-7, -8, -16
8-OH	2.09 d (10.8)		H-8	C-8

a n.o. = not observed.

**Table 3:** Cytotoxic data of compounds **1** and **2**.

Compounds	Cell lines IC_50_ (μM)	

	HL-60	MOLT-4
**1**	NA	NA
**2**	NA	14.42
**Doxorubicin**^a^	0.06	0.02

a Doxorubicin was used as a positive control.

NA = not active at 20 μM for 72 h.
